# A Rare Incidence of Splenic Artery Aneurysm and Hypersplenism

**DOI:** 10.7759/cureus.54280

**Published:** 2024-02-16

**Authors:** Nabil M Azmi, Lenny Suryani Safri, Nurafdzillah Abdul Rahman, Diana Melissa Dualim, Soma Chandrakanthan

**Affiliations:** 1 Department of Surgery, Faculty of Medicine, The National University of Malaysia, Kuala Lumpur, MYS

**Keywords:** embolisation, interventional radiology, splenectomy, hypersplenism, splenic aneurysm, splenic artery

## Abstract

A 31-year-old woman with Child's B liver cirrhosis with portal hypertension and splenomegaly presented with a one-month history of abdominal pain. A physical examination confirmed splenomegaly. A blood investigation revealed a low white blood cell (WBC) and platelet count. Computed tomography (CT) revealed a splenic artery aneurysm at the distal splenic artery measuring 3.4 x 3.4 x 4.3 cm (AP x W x CC) with thrombus and splenic infarction. A successful angiographic embolisation was performed without immediate complications. The abdominal pain symptoms resolved, leading to the patient's discharge from the ward on the third day post-embolisation. Follow-up at the surgical outpatient clinic indicated the patient remained asymptomatic, and repeated blood counts showed improvement in both WBC and platelet counts. Furthermore, follow-up CT scans demonstrated a reduction in spleen size, indicating positive outcomes and a favourable response to the intervention.

## Introduction

Visceral artery aneurysm and pseudoaneurysm (VAAP) are uncommon but potentially life-threatening conditions. Among these, splenic artery aneurysm (SAA) ranks as the third most prevalent type of VAAP. SAA is defined as the dilation of the artery by more than 50% compared to the normal vessel diameter, with a true aneurysm involving all layers of the artery [1]. Risk factors for SAA include pregnancy, portal hypertension, and autoimmune diseases. The reported incidence of SAA is less than 1% of the population, with the majority being asymptomatic [2].

In this case report, we present the instance of a woman with SAA and hypersplenism who initially complained of vague left-sided abdominal pain, coinciding with underlying Child's B liver cirrhosis. While the need for intervention was clear in this case, the optimal treatment modality remains a subject of debate. This case demonstrates that embolisation proved to be effective not only for addressing SAA but also in the treatment of hypersplenism.

## Case presentation

This is a case of a 31-year-old woman with a history of Child's B liver cirrhosis, portal hypertension, splenomegaly, and a blood investigation showing a low WBC of 1.9 x 10^9 cells/L and low platelet counts of 54 x 10^9 cells/L. She presented with a one-month history of abdominal pain, describing it as dull and localised in the left hypochondriac region. Vital signs were within normal limits, and no fever was recorded. Abdominal examination revealed no stigmata of chronic liver disease but Hackett's Grade 2 splenomegaly. As stated, relevant blood counts indicate leucopaenia and thrombocytopaenia consistent with hypersplenism.

**Figure 1:**
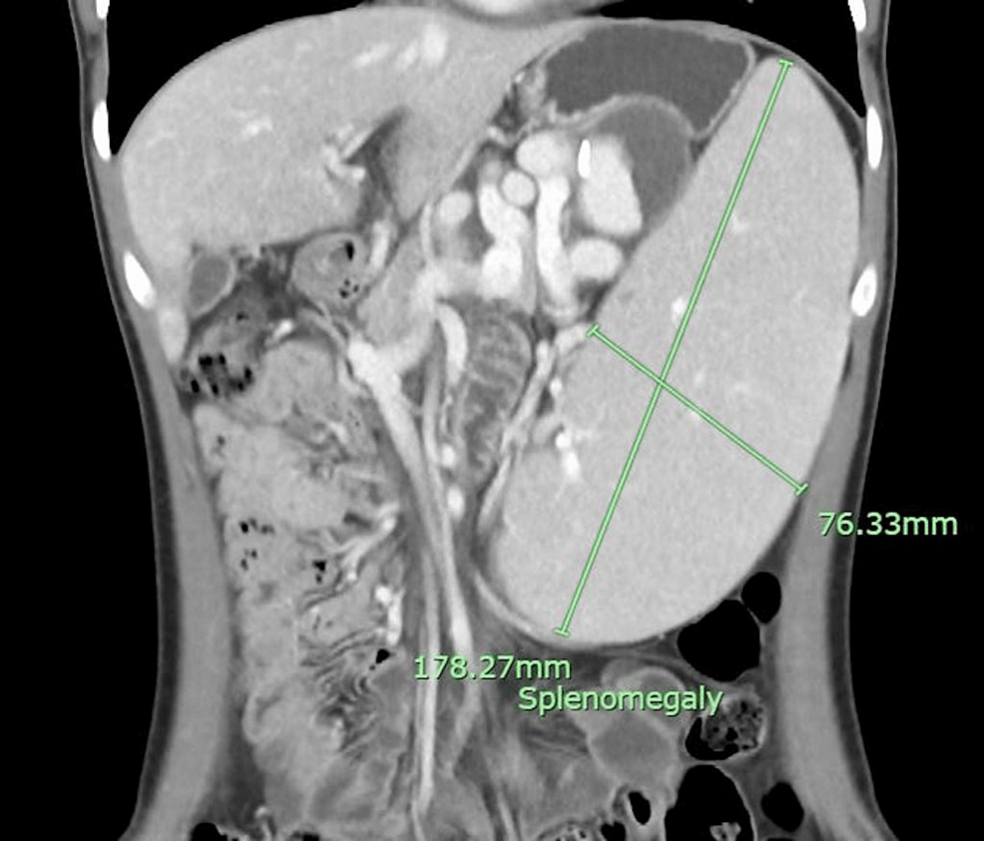
Computed tomography (CT) scan (coronal view) showing splenomegaly

**Figure 2:**
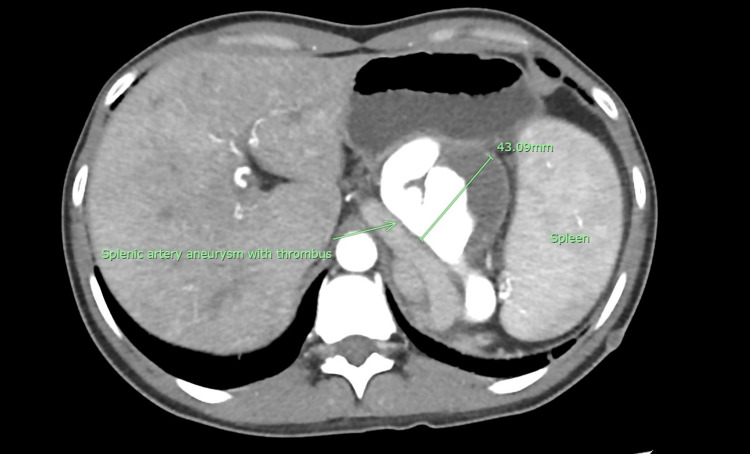
Computed tomography (CT) angiogram (axial view) showing splenic artery aneurysm (SAA) with thrombus before embolisation

Upon admission, a contrast-enhanced computed tomography (CT) of the abdomen was scheduled, revealing a saccular-type SAA at the mid to distal splenic artery measuring 3.4 x 3.4 x 4.3 cm (AP x W x CC) with thrombus and splenic infarction as depicted in Figures 1-2.

A decision was made to perform angiographic coiling of the SAA. The femoral artery on the right side was punctured to establish vascular access for embolization. Following the identification of the SAA, fibered angiocoils of different sizes along with 2 ml of lipiodol were utilised as the embolisation material. Lipiodol, an iodine-based embolisation material, helped to delineate the vessel architecture better. The procedure was considered successful upon visual confirmation of thrombosis within the SAA.

**Figure 3:**
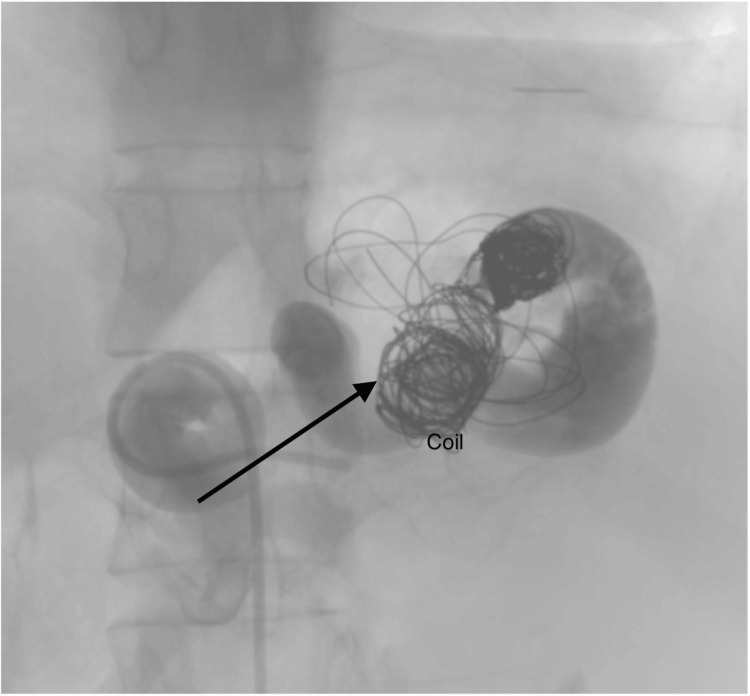
Angiogram showing post-coiling embolization. The arrow indicates the distal end of the aneurysm. No contrast flow to the spleen after successful embolisation

The patient was monitored in the surgical ward, experiencing initial left-sided abdominal pain that was successfully relieved with analgesia. Throughout her stay, vital signs remained stable. A follow-up CT angiogram was conducted to assess the splenic artery post-coiling. The imaging revealed a thrombosed splenic artery, evident from the opacification of the splenic artery branch, as illustrated in Figure 3. Despite this finding, the patient remained asymptomatic and was discharged on the same day.

**Figure 4:**
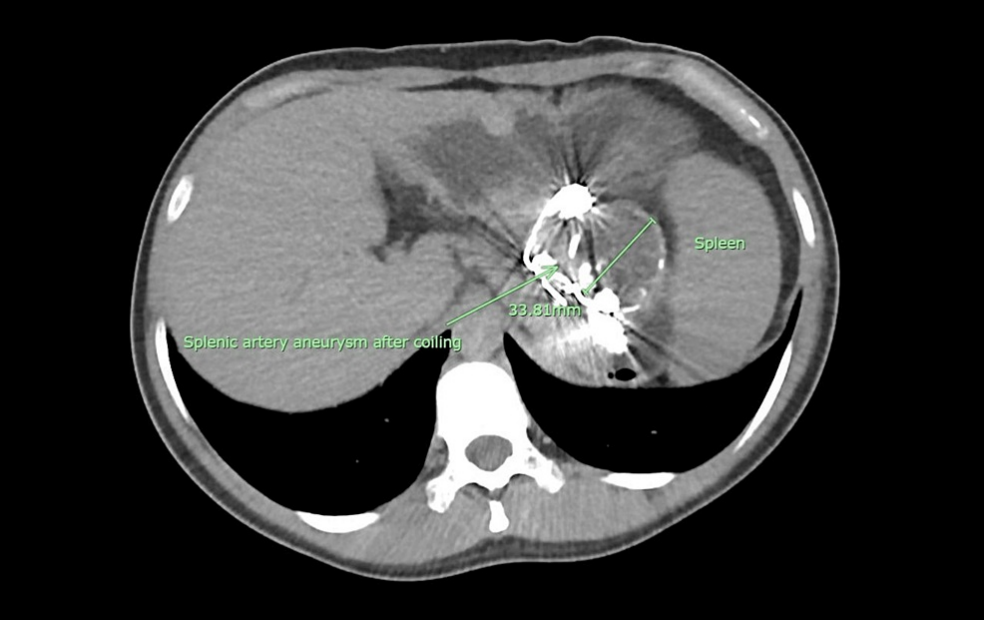
Computed tomography (CT) scan depicting thrombosed splenic artery aneurysm (SAA) with coils within

During a follow-up visit to the vascular clinic, there was a notable improvement in the patient's blood counts. The follow-up peripheral blood parameters indicated an increase in WBC from 1.9 to 5.0 x 10^9 cells/L and platelet counts from 54 to 209 x 10^9 cells/L. Another follow-up CT of the abdomen demonstrated a reduction in spleen size from 10.8 x 7.4 x 18.6 to 9.3 x 5.4 x 16.1 cm (AP x W x CC), as shown in Figure 4. These positive developments suggest a favourable response to the intervention and a positive trajectory in the patient's recovery.

## Discussion

The occurrence of SAA is exceptionally uncommon, with a reported prevalence of 0.7% in the general population. However, autopsy studies suggest it could be as high as 10%, indicating that a significant number of cases may go undetected. Interestingly, SAA is observed to be four times more frequent in females than in males, particularly in pregnant women. This discrepancy is attributed to hemodynamic and hormonal shifts during pregnancy and is distinct from other types of visceral artery aneurysms. While the specific cause of the aneurysm in this case is uncertain, it may be linked to increased blood flow through the splenic artery. Larger portosystemic shunts cause a rise in portal blood inflow volume, which seems to contribute to dilatation of the splenic artery [3-5].

Instances of SAA accompanied by hypersplenism are infrequent, and there appears to be a shortage of documented cases and subsequent treatment approaches. The increasing availability of CT scans and improved radiological interpretation, often conducted for other medical reasons, have contributed to the growing identification of SAA cases. Treatment is recommended for symptomatic patients, such as the one in this case. Conservative management involving serial CT scans over six to 12 months is suggested. Intervention becomes necessary if the diameter exceeds 30 mm, as it poses a significant risk of rupture. However, the evidence for managing SAAs between 20 mm and 30 mm in diameter is not conclusive [6]. Typically, SAAs exhibit a saccular shape, with aneurysmal dilation commonly occurring at the mid and distal portions of the splenic artery, as reported by D. Sibalis et al. In the described case study, successful treatment involved transcatheter embolisation to halt the bleeding from the ruptured SAA [7].

The proposed treatments for SAA encompass surgical options like splenectomy for the prevention of aneurysmal rupture, ligation of the splenic artery, and endovascular methods such as stent grafts or angioembolization. In this specific case, the preferred approach is the embolisation of the splenic artery using coils. This preference arises because the patient is not a suitable candidate for splenectomy due to her Child’s B liver cirrhosis, which increases the risk of perioperative bleeding given her low platelet count. Additionally, the patient is susceptible to various post-operative complications, including surgical site infection and liver failure.

Moreover, the incidence of overwhelming post-splenectomy sepsis poses a significant life-threatening risk. Without a spleen, the patient would require antibiotic coverage for any invasive procedure, as mandated by local guidelines in the future. This consideration emphasises the importance of selecting an alternative treatment method, such as embolisation, to address the SAA while mitigating the associated risks linked to the patient's medical condition.

Various surgical options are available and considered in specific cases of SAA. These options include splenectomy, splenic artery ligation, and aneurysmal excision with reanastomosis. However, these surgical interventions are typically reserved for selected cases and the available expertise of the surgeons. For instance, embolisation may not be the preferred choice when it is challenging to perform or if the aneurysm is located in close proximity to the splenic hilum [8]. The decision on the most appropriate treatment is also influenced by the fitness of the patient to undergo surgery, emphasising the importance of patient selection in determining the optimal treatment approach.

This case illustrates the feasibility of angiographic coiling for treating SAA and hypersplenism in a single procedure. This approach presents lower morbidity compared to surgery and, importantly, allows for spleen preservation [9]. The patient remained asymptomatic, and follow-up with repeated CT scans indicated a reduction in spleen size with infarction along with a recovery in WBC and platelet counts. These findings align with a study by He et al. [7], which concluded that splenic artery embolisation yields better long-term outcomes spanning from two weeks of follow-up to four years. The presence of embolisation material induces thrombosis of the splenic artery and has caused hypoperfusion to the spleen, promoting splenic atrophy. A follow-up CT scan, however, does not indicate the progression of splenic infarction. The method is considered safe and efficacious, with a low incidence of post-embolisation syndrome and splenic abscess.

However, a study conducted by Li et al. [8] appears contradictory regarding recovery from hypersplenism after splenic artery embolization. The author suggested that while splenic artery embolisation in patients with SAA may lead to a return to normal spleen size, it may not necessarily result in a full recovery of WBC and platelet counts. Despite this discrepancy, splenic artery embolisation remains a viable and effective treatment method with a favourable safety profile.

## Conclusions

Angiographic coiling emerges as the preferred treatment option for symptomatic SAAs in patients with liver cirrhosis. This approach not only results in a reduction in spleen size but also demonstrates improvements in WBC and platelet counts associated with hypersplenism. Importantly, angiographic coiling is considered a safe option, characterised by a low morbidity rate.
